# Genome-Wide Identification and Expression Profiling Analysis of the Trihelix Gene Family Under Abiotic Stresses in *Medicago truncatula*

**DOI:** 10.3390/genes11111389

**Published:** 2020-11-23

**Authors:** Xiqiang Liu, Han Zhang, Lin Ma, Zan Wang, Kun Wang

**Affiliations:** 1 College of Grassland Science and Technology, China Agricultural University, Beijing 100193, China; xiqiangliu003@126.com (X.L.); hanzhang003@126.com (H.Z.); zanwang@cau.edu.cn (Z.W.); 2 Institute of Animal Science, Chinese Academy of Agricultural Sciences, Beijing 100193, China; malin@caas.cn

**Keywords:** trihelix transcription factor family, *Medicago truncatula*, GT, expression profile, drought and salt stress

## Abstract

The trihelix transcription factor (GT) family is widely involved in regulating plant growth and development, and most importantly, responding to various abiotic stresses. Our study first reported the genome-wide identification and analysis of GT family genes in *Medicago truncatula*. Overall, 38 trihelix genes were identified in the *M. truncatula* genome and were classified into five subfamilies (GT-1, GT-2, SH4, GTγ and SIP1). We systematically analyzed the phylogenetic relationship, chromosomal distribution, tandem and segmental duplication events, gene structures and conserved motifs of MtGTs. Syntenic analysis revealed that trihelix family genes in *M. truncatula* had the most collinearity relationship with those in soybean followed by alfalfa, but very little collinearity with those in the maize and rice. Additionally, tissue-specific expression analysis of trihelix family genes suggested that they played various roles in the growth and development of specific tissues in *M. truncatula*. Moreover, the expression of some *MtGT* genes, such as *MtGT19*, *MtGT20*, *MtGT22*, and *MtGT33*, was dramatically induced by drought, salt, and ABA treatments, illustrating their vital roles in response to abiotic stresses. These findings are helpful for improving the comprehensive understanding of trihelix family; additionally, the study provides candidate genes for achieving the genetic improvement of stress resistance in legumes.

## 1. Introduction

Transcription factors (TFs) are a kind of DNA binding protein, that play pivotal roles in plant growth and development, as well as in response to environmental stresses [1,2]. TFs regulate the expression of target genes by binding to specific *cis*-elements of the gene promoter region or binding with other TFs [3]. Currently, trihelix TF family members have attracted more attention; they feature a typical helix–loop–helix–loop–helix structure with a core sequence of 5′-G-Pu-(T/A)-A-A-(T/A)-3′ in their DNA-binding domain [4,5]. Because this domain can specifically bind to GT elements required for light response in a DNA sequence, it is also called the GT family [6]. The conserved domain of trihelix is similar to the individual repeats of the MYB family in sequence; therefore, it is generally thought to be derived from *MYB*-like genes [5].

The first discovered trihelix gene was the GT-1 transcription factor in pea (*Pisum sativum*) [7]. Subsequently, orthologous genes were identified in tobacco and *Arabidopsis thaliana* [8,9]. Early researches on GT genes had been focused on the light response regulation [10,11,12]. In recent years, the biological functions of several trihelix family genes have been discovered, indicating that they are widely involved in fine-tuning a range of specialized developmental processes including flowers, trichomes, stomata, embryos and seeds, and most importantly, responding to various abiotic stresses in plants [12,13,14,15,16,17,18]. Based on the phylogenetic relationship and characteristics of the conserved domain, the trihelix genes family is divided into five subfamilies: GT-1, GT-2, GTγ, SH4, and SIP1. Park et al. found that *AtGT-3b* was rapidly induced by salt stress and could interact with the GT-1 *cis*-element of the *SCaM-4* promoter in *Glycine max*, thereby responding to various environmental stresses [18]. The GT-2 LIKE 1 (*AtGTL1*) can improve water use efficiency and drought tolerance by trans-repressing the expression of the *STOMATAL DENSITY AND DISTRIBUTION1* (*SDD1*) gene, encoding a subtilisin-like serine protease that negatively regulates stomatal generation in *Arabidopsis* [15]. Also in wheat, *TaGT2L1D*, the homologous gene of *AtGTL1*, plays a similar role and affects floral organ development [19]. The expression levels of three GTγ clade genes in rice (*OsGTγ-1*, *OsGTγ-2,* and *OsGTγ-3)* increased significantly under salt stress; moreover, the *OsGTγ-1* gene could respond to drought and cold stress [20]. Lin et al. demonstrated that *Shattering1* (*SHA1*), encoding a member of the trihelix SH4 subfamily, plays an important role in the activation of cell separation in the shattering process of rice seeds [17].

*M. truncatula* is a diploid plant with a relatively small genome, adopted as a model species for legumes genetics and functional genomics research [21,22]. Particularly, its genome has a high similarity to that of alfalfa (*Medicago sativa*), the most widely cultivated economic forage plant in the world; hence, studies on *M. truncatula* can provide important genetic reference information for alfalfa. At present, trihelix genes have been systematically identified and analyzed in many plants, including *Arabidopsis* [12], *Oryza sativa* [23], *Solanum lycopersicum* [24], *Brassica rapa* [25], *Gossypium arboreum* [26], *G. max* [27], *Chrysanthemum morifolium* [28], *Triticum aestivum* [29] and *Populus trichocarpa* [30]. However, information and functional analysis of the trihelix family in *M. truncatula* has not yet been performed. Because trihelix family genes are widely involved in plant growth and development and respond to various abiotic stresses, identifying and analyzing the expression characteristics of trihelix family members in *M. truncatula* is of great significance.

In this study, we performed a systematic genome-wide investigation of the trihelix gene family in *M. truncatula*. Overall, 38 *MtGT* genes were identified, and their phylogenetic relationship, chromosomal localization, gene structures, and motif compositions were analyzed in detail. We demonstrated the collinearity of *MtGT* genes with *A. thaliana*, *G. max*, *Zea mays*, *O. sativa* and *M. sativa* genomes. Additionally, we performed tissue-specific expression analysis of *MtGT* genes in six tissues (blade, bud, nodule, flower, root, and seedpod) and verified by real-time quantitative PCR (qRT-PCR). Moreover, we explored and validated the expression profiling of *MtGT* genes in response to drought and salt stresses. Therefore, this study is helpful to understand the trihelix family more comprehensively and provides a reference functional gene resource, particularly for alfalfa, for the genetic improvement of stress resistance in legumes.

## 2. Materials and Methods

### 2.1. Plant Materials and Treatments

*M. truncatula* (cv. Jemalong A17) seeds were sterilized in 75% ethanol for 5 min, rinsed with sterile water five times, and then placed on the moistened filter paper in Petri dishes. They were subsequently cultured in a growth cabinet at 25 °C. For the tissue-specific expression analysis of *MtGT* genes, 7-day-old seedlings were transferred into the mixture of peat soil and vermiculite (1:1, *v/v*) for individual pot cultivation (18 cm inner diameter, 20 cm height). Potted seedlings grew in a greenhouse (the temperature is 25 °C with a 16/8 h light/dark photoperiod, relative humidity of 35–40%, and photon flux density of 450 μmol m^−2^·s^−1^). Roots, stems, blades, buds, flowers and seedpods were collected at pod stage from three individual plants. For the expression analysis of *MtGT* genes response to different treatments, the 7-day-old seedlings were transferred into the flasks with 1/2 MS liquid medium and grew in a controlled growth chamber under 16/8 h light/dark regime at 25 °C. Ten days later, the plants with the fourth blade expanded were watered with 15% PEG6000, 200 mmol·L^−1^ NaCl, and 1 mmol·L^−1^ ABA, respectively. The blades were collected at 0 h, 2 h, 24 h and 72 h with a triplicate. All samples were immediately frozen in liquid nitrogen and stored at −80 °C for RNA extraction.

### 2.2. Identification of the Trihelix Genes in M. truncatula

The *M. truncatula* genome information was downloaded from *M. truncatula* Genome Database (MTGD, http://www.medicagogenome.org/downloads). Based on two BLASTp methods, the 35 trihelix protein sequences of *Arabidopsis* retrieved from Plant Transcription Factor Database (PlantTFDB, http://planttfdb.cbi.pku.edu.cn/) [31] were used as query to obtain the possible trihelix proteins in *M. truncatula* genome by BLASTp search with a cutoff E-value of 1.0 × 10^−10^. Furthermore, the Hidden Markov Model (HMM) profile (PF13837) was used to identify the putative trihelix domain in Pfam database (http://pfam.xfam.org/) [32]. The Conserved Domain Database (CDD) of NCBI (https://www.ncbi.nlm.nih.gov/cdd/) [33] and SMART (http://smart.embl-heidelberg.de) [34] were used to confirm the trihelix proteins in all the candidate proteins. The basic physical and chemical characteristics of 38 trihelix proteins, such as the molecular weight (MW), isoelectric point (pI) and grand average of hydropathicity (GRAVY), were determined on ExPASy (http://web.expasy.org/protparam/) [35]. The subcellular localization of MtGTs were predicted on the Plant-mPLoc website (http://www.csbio.sjtu.edu.cn/bioinf/plant-multi/) [36].

### 2.3. Chromosomal Distribution and Gene Duplication Events Analysis

The chromosomal location information of 38 trihelix genes were obtained from the *M. truncatula* genomic annotation file GFF3 (general feature format) that was downloaded in MTGD. The TBtools software [37] was used to draw the chromosomal distribution image of *MtGT* genes. The detection and identification of the gene duplication events in *MtGT* genes were performed using multiple collinear scanning toolkits (MCScanX) [38] with E-value set to 10^−5^.

### 2.4. Gene Structure and Conserved Motifs Analysis

Gene structure was constructed to visualize the exon-intron of *MtGT* genes based on the CDS and the corresponding full-length sequence by using TBtools software [37]. The MEME tool (http://meme-suite.org/tools/meme) [39] was used to analyze the conserved motifs of MtGT proteins and the relative parameters were set to the motif width as 15–50 amino acid (aa) and the number of motifs as 10.

### 2.5. Phylogenetic and Collinearity Analysis of MtGT genes

The full-length amino acid sequences of the *M. truncatula* and *Arabidopsis* trihelix family proteins were aligned using MUSCLE, and were visualized by Jalview 2 [40]. The MEGA X [41] was used to construct the unrooted phylogenetic tree by Neighbor-Joining (NJ) method with a bootstrap value of 1000 replicates. The phylogenetic tree was illustrated using online tool EvolView (http://www.evolgenius.info/evolview/) [42]. To analyze syntenic relationships of the trihelix family genes among the *M. truncatula* and *Arabidopsis*, *G. max*, *Z. mays*, *O. sativa* and *M. sativa* genomes, MCScanX was used with default settings [38] and was visualized by TBtools software [37].

### 2.6. Tissue-Specific Expression and Abiotic Stress Expression Analysis

The relative FPKM value (fragments per kilobase of transcript per million fragments mapped) of 36 *MtGT* genes in six tissues (blade, bud, nodule, flower, root, seedpod) of RNA-seq data were retrieved from MTGD (http://www.medicagogenome.org/) and the heat map of hierarchical clustering was visualized using TBtools software [37]. 33 *MtGT* genes expression microarray data of 28-day-old seedlings under drought (40–45% soil water content) and salt (200 mM NaCl) treatments were obtained from the *M. truncatula* Gene Expression Atlas (MtGEA) (https://mtgea.noble.org/v3/) Web Server [43], and visualized for heat map using TBtools software [37].

### 2.7. Expression Analysis of the MtGT Genes by Real-Time qPCR

Total RNAs were extracted with the Eastep^®^ Super Total RNA Extraction Kit (Promega, Beijing, China) following the manufacturer’s instructions and then EasyScript^®^ All-in-One First-Strand cDNA Synthesis SuperMix for qPCR (TransGen, Beijing, China) was used to synthesize the cDNA. Quantitative real-time PCR (qRT-PCR) was conducted according to the instructions of 2×RealStar Green Fast Mixture with ROX II (GenStar, Beijing, China) on an ABI QuantStudio^TM^ 7 Flex RT-PCR system (Applied Biosystems, Foster City, CA, USA). The gene-specific primer sequences for qRT-PCR determination are provided in Table S9. *MtActin* was used as the internal control. Three technical repetitions for each sample, and the relative expression data were calculated according to the 2^−ΔΔCT^ method [44].

## 3. Results

### 3.1. Identification of MtGT Genes in M. truncatula

In this study, 38 non-redundant trihelix genes were identified in the *M. truncatula* genome through two BLAST methods based on the known trihelix protein sequences of *Arabidopsis*, and both Pfam and CDD databases confirmed the presence of trihelix domain. Subsequently, 38 *MtGT* genes were named *MtGT1* to *MtGT38* according to their order on the chromosomes (Table 1). The predicted physical and chemical properties of MtGT proteins, including protein length, molecular weight (MW), isoelectric point (pI), and grand average of hydropathicity (GRAVY) are shown in Table 1. The length of MtGT proteins varied from 189 (MtGT-2) to 1223 (MtGT-15) amino acids (aa), with a MW range of 21.31–140.86 kDa. The average length of these proteins was 428 aa, and their average MW was 48.69 kDa. The pI ranged from 4.48 (MtGT-17) to 9.73 (MtGT-24). The GRAVY ranged from −0.400 (MtGT-19) to −1.395 (MtGT-17), and the average GRAVY was 0.953. Subcellular localization prediction revealed that most MtGT proteins are located in the nucleus, which is consistent with the role of TFs. Interestingly, the remaining four proteins (MtGT-11, MtGT-12, MtGT-13, and MtGT-20) are located in the chloroplast, which may be related to photosynthesis. The nucleic acid sequences of *MtGT* genes and encoded amino acid sequences are provided in Table S1.

### 3.2. Phylogenetic Analysis and Classification of MtGT Genes

**Figure 1:**
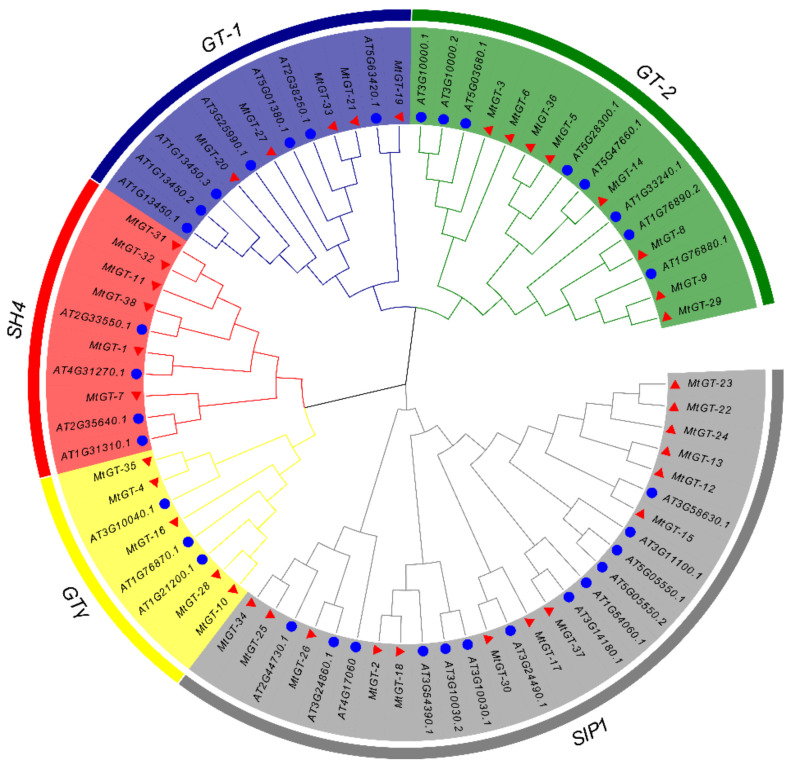
Phylogenetic tree of trihelix proteins in *M. truncatula* and *A. thaliana*. The branches with different colors represent different subfamilies. The trihelix proteins of *M. truncatula* and *A. thaliana* are marked as red triangles and blue circles, respectively.

To investigate the molecular evolution and phylogenetic relationship of the trihelix family in *M. truncatula*, multiple sequences alignment of 35 *A. thaliana* trihelix proteins [12,31] and 38 MtGT proteins was performed (Figure S1) and the unrooted phylogenetic tree was constructed (Figure 1). A total of 73 trihelix proteins were classified into five clades (GT-1, GT-2, SH4, GTγ and SIP1), consistent with the previous studies on *Arabidopsis* [14] and other species, such as soybean [24], rice [17], tomato [21], and wheat [26]. Among these, SIP1 with 14 MtGT family members was the largest cluster, whereas GT-1 and GTγ were the smallest subfamily with 5 MtGT family members each. GT-2 and SH4 clades contained eight and six MtGT members, respectively. These results were similar to the genes distribution of different subgroups in *Arabidopsis* and rice [12,23].

### 3.3. Chromosomal Distribution and Gene Duplication Events of MtGT Family

**Figure 2:**
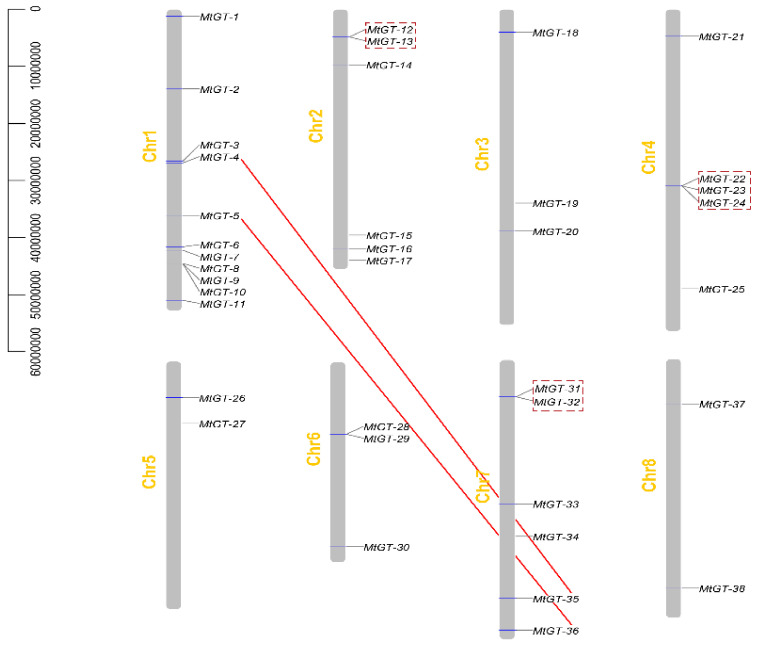
Chromosomal distribution and duplication events of *MtGT* genes. The chromosome number is labelled on the left of each chromosome. Red boxes indicate tandem duplication, and red lines indicate segmental duplication.

As shown in the chromosome map (Figure 2), 38 *MtGT* genes were located unevenly on eight chromosomes. Chromosome 1 contained most genes (11) of trihelix family, whereas chromosomes 5 and 8 had the least number of genes (2). To clarify the molecular evolution of the MtGT family, gene duplication events including tandem and segmental duplication were analyzed. In this study, we identified three groups of *MtGT* genes with tandem duplication events (*MtGT-12*, *MtGT-13*; *MtGT-22*, *MtGT-23*, *MtGT-24*; and *MtGT-31*, *MtGT-32*), and segmental duplication existed in two pairs of genes (*MtGT-4*, *MtGT-35* and *MtGT-5*, *MtGT-36*) (Figure 2). Tandem and segmental duplication events facilitated divergence of genes with novel functions, which play a key role in promoting the evolution and expansion of gene families to help plants in adapting to various environmental conditions [25,45].

### 3.4. Gene Structural Characteristics and Conserved Motifs Compositions of MtGT Genes

**Figure 3:**
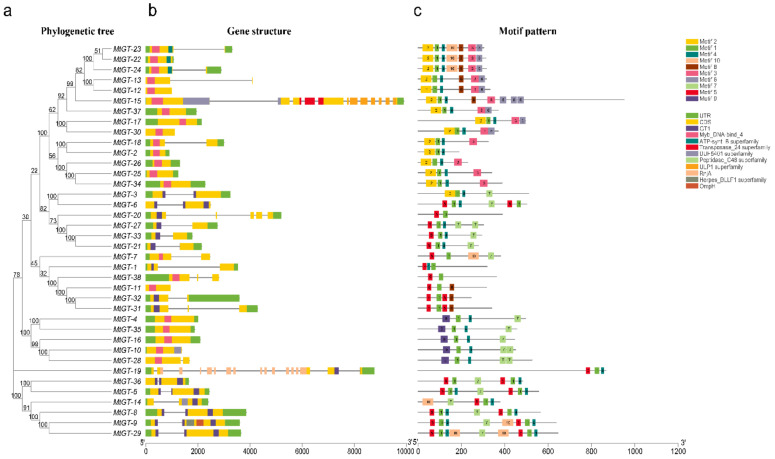
Gene structures and conserved motifs of the *MtGT* genes. (**a**) The phylogenetic tree was constructed based on the amino acid sequences of *M. truncatula* trihelix proteins. (**b**) Gene structures of *M. truncatula* trihelix genes. Green boxes indicate untranslated 5′- and 3′-regions, yellow boxes indicate exons, and gray lines indicate introns. The trihelix domains are labelled with dark blue boxes (GT1) and pink boxes (MYB DNA-binding). (**c**) The motif compositions of *M. truncatula* trihelix proteins. The motif 1-motif 10 are displayed in different colored boxes. The protein length can be estimated using the scale at the bottom.

The features of the *MtGT* genes structure are shown in Figure 3b, including the distribution of exons and introns and the types of protein domains. The most *M. truncatula* trihelix genes grouped in the same subfamily shared similar exon/intron organizations and domains. All *MtGT* genes contained the GT domain or MYB DNA-binding domain, in which members belonging to the SIP1 or GTγ clade contained the MYB DNA-binding domain, and most *MtGT* genes in GT-1, GT-2, and SH4 clades possessed the GT domain. Further, the majority of *MtGT* family members had very few introns (0–2) and 14 of them had no intron. The GTγ subfamily members (*MtGT4*, *MtGT10*, *MtGT16*, and *MtGT35*) had no intron except in *MtGT28*, which coincided with the findings of previous studies of trihelix families in *B. rapa* [25], wheat [29] and *Fagopyrum tataricum* [46]. In the remaining genes, *MtGT19* had most introns (16), *MtGT-15,* which was the longest trihelix gene, had 11 introns, and *MtGT20* contained four introns (Figure 3b). To further analyze the diversity of MtGT proteins, the MEME search tool was used to identify 10 conserved motifs (motif 1–motif 10) shown in Figure 3c, and the detailed sequence of each motif is provided in Table S2. Motif 1 was presented in all MtGT proteins. Motif 5 existed almost in all GT-1, GT-2, and SH4 clades. Among them, *MtGT-5*, *MtGT-6*, *MtGT-8*, *MtGT-9*, *MtGT-29*, *MtGT-31*, *MtGT-32*, and *MtGT-36* had two motif 5, of which most belonged to clade GT-2. In addition, GTγ clade genes featured motif 9 at their N-terminal, and all SIP1 subfamily genes contained motif 2 at their N-terminal and featured motif 6 at C-terminal.

### 3.5. Evolutionary and Collinearity Analysis within MtGT Genes and Several Species

**Figure 4:**
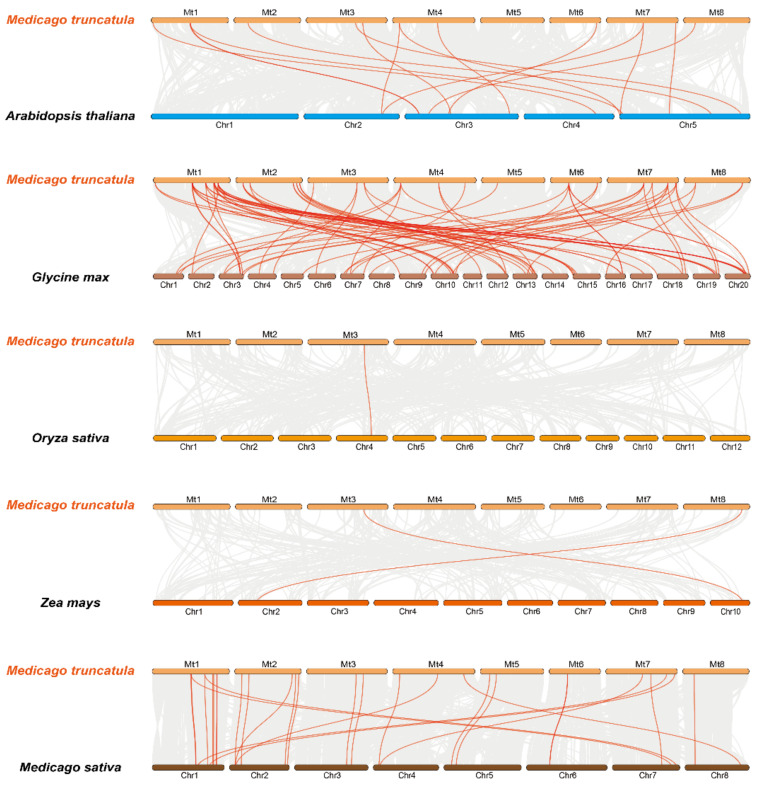
Syntenic analysis of the *MtGT* genes between *M. truncatula* and five representative plant species (*A. thaliana*, *G. max*, *Z. mays*, *O. sativa* and *M. sativa*).

To further understand the collinearity of the *M. truncatula* trihelix family, we constructed five comparative syntenic diagrams between *M. truncatula* and the representative species including three dicotyledonous plants (*Arabidopsis*, soybean, and alfalfa) and two monocotyledonous (rice and maize) (Figure 4). The details are provided in Table S3. *MtGT* genes displayed syntenic relationships in different degrees with five species; they had the most collinearity relationship with soybean, followed by alfalfa, and had very little collinearity relationship with the maize and rice. A total of 25 and 22 *MtGT* genes showed syntenic relationships with soybean and alfalfa, respectively. However, only one and two genes had collinearity relationships with rice and maize, respectively. Clearly, the study of *MtGT* family genes can provide a more valuable gene functional reference for legume crops.

### 3.6. Expression Patterns of MtGT Genes in Different Tissues

**Figure 5:**
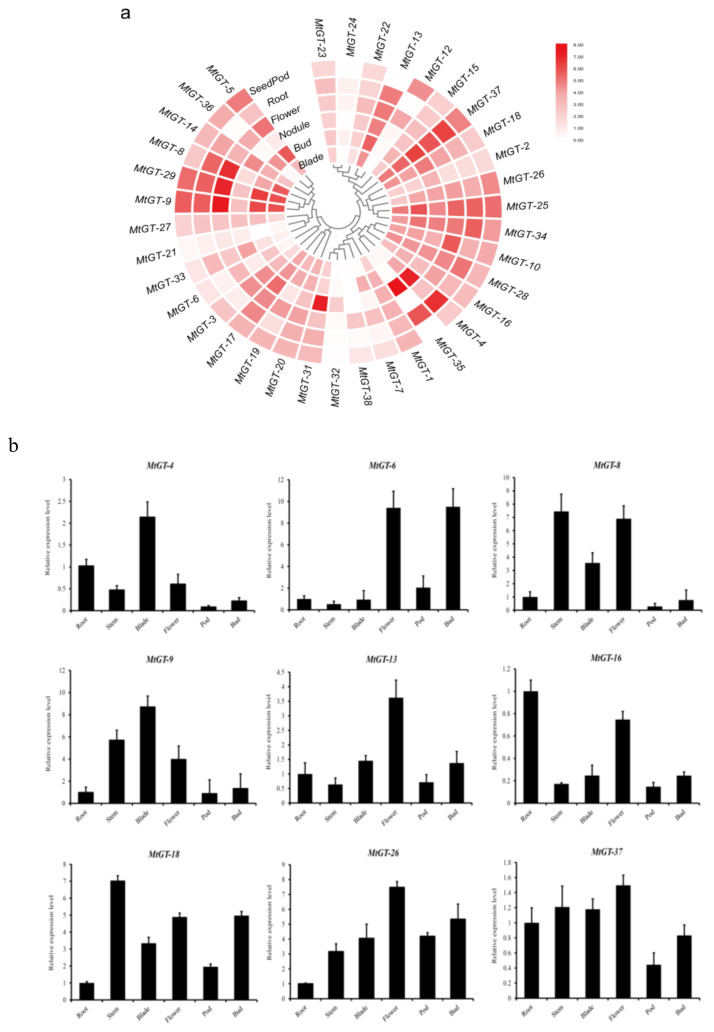
Tissue-specific expression characteristics of *MtGT* genes. (**a**) The expression heatmap of 36 *MtGT* genes in six tissues (root, blade, bud, flower, seedpod and nodule) from RNA-seq data. (**b**) The expression patterns of 9 *MtGT* genes in six tissues (root, stem, blade, flower, bud and seedpod) were validated by real-time qPCR. Expression levels were normalized using *MtActin* as the internal control and error bars indicated standard deviation among three biological replicates.

The tissue-specific expression data of *MtGT* genes in six tissues by RNA-seq were retrieved from MTGD (http://www.medicagogenome.org/) (Table S4), including blade, bud, nodule, flower, root, and seedpod, which are shown with a heat map in Figure 5a. Except for the expression data of *MtGT-11* and *MtGT-30*, which were not found, the other 36 *MtGT* genes had different expression levels in six tissues. Among them, some *MtGT* genes were highly expressed in specific tissues, *MtGT-8*, *MtGT-9*, and *MtGT-29* were expressed at relatively high levels in roots, flowers, and blades; *MtGT12* was specifically expressed in flowers and seedpods; *MtGT4* and *MtGT35* were expressed at high levels in roots and nodules; and *MtGT31* and *MtGT32* were only expressed highly in nodules. However, there were several *MtGT* genes whose expression levels were similar in six tissues, such as *Mt-GT1*, *MtGT-27*, *MtGT-20*, *MtGT-15*, *MtGT-22,* and *MtGT-23*. In addition, the expression levels of *MtGT21* and *MtGT24* in various tissues were very low (Figure 5a). The expression levels of nine selected *MtGT* genes were further verified through qRT-PCR in the six tissues (root, stem, blade, flower, bud and seedpod) (Figure 5b; Table S5). The results demonstrated that tissue expression levels of most selected *MtGT* genes were consistent with the RNA-seq data from MTGD except *MtGT4*, suggesting that *MtGT* family members play various roles in specific tissues during the growth and development of *M. truncatula*.

### 3.7. Expression Profiling Analysis of MtGT Genes in Response to Abiotic Stress

**Figure 6:**
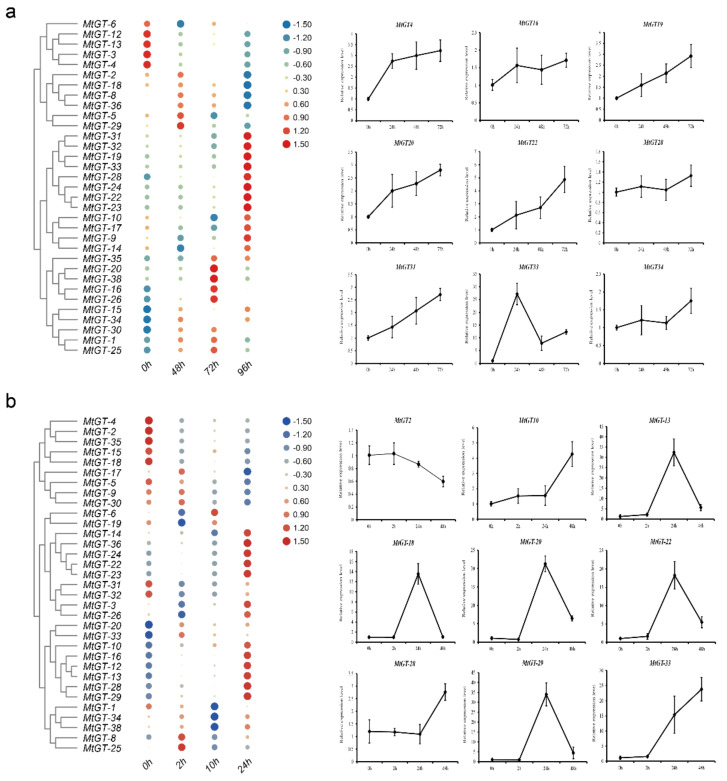
Expression profiles of the *MtGT* genes in response to abiotic stresses. A total of 33 *MtGT* genes chip expression data of 28-day-old seedlings under drought (40–45% soil water content) (**a**) and salt (200 mM NaCl) treatments (**b**). The relative expression levels were -log^2^ transformed and visualized by heat map. The colors vary from blue to red, and circles from small to large represent the scale of the relative expression levels. The expression patterns of nine *MtGT* genes under drought and salt treatments were validated by qRT-PCR. Expression data were normalized using *MtActin* as the internal control and error bars indicate standard deviation among three biological replicates.

Recent studies have indicated that trihelix genes play crucial roles in plants response to abiotic stresses. Based on the *M. truncatula* Gene Expression Atlas (https://mtgea.noble.org/v3/), we obtained the 33 *MtGT* gene chips expression data in the blades of 28-day-old seedlings under drought and salt treatments (Table S6). Through the differential expression analysis of these genes, we found that 12 *MtGT* genes (*MtGT-31*, *MtGT-32*, *MtGT-19*, *MtGT-33*, *MtGT-28*, *MtGT-24*, *MtGT-22*, *MtGT-23*, *MtGT-10*, *MtGT-17*, *MtGT-9*, and *MtGT-14*) were significantly up-regulated and 5 *MtGT* genes (*MtGT-3*, *MtGT-4*, *MtGT-6*, *MtGT-12*, and *MtGT-13*) were significantly down-regulated under drought stress for 96 h (Figure 6a). Other genes (*MtGT-20*, *MtGT-38*, *MtGT-16*, and *MtGT-26*) were significantly up-regulated for 72 h of drought treatment. Additionally, the salt stress expression analysis of *MtGT* genes revealed that 13 *MtGT* genes (*MtGT-14*, *MtGT-36*, *MtGT-24*, *MtGT-22*, *MtGT-23*, *MtGT-3*, *MtGT-26*, *MtGT-10*, *MtGT-16*, *MtGT-12*, *MtGT-13*, *MtGT-28*, and *MtGT-29*) were significantly up-regulated, and 8 *MtGT* genes (*MtGT-4*, *MtGT-2*, *MtGT-35*, *MtGT-15*, *MtGT-18*, *MtGT-5*, *MtGT-31*, and *MtGT-32*) were significantly down-regulated under 24-h salt stress (Figure 6b). However, *MtGT-8* and *MtGT-25* were significantly up-regulated under 2 h of salt stress, and then their expression levels decreased. *MtGT-6* was significantly up-regulated under 10-h salt stress. Among these genes, many genes could respond to both drought and salt stress. The expression profiles of nine *MtGT* genes under drought and salt treatments were validated by qRT-PCR (Figure 6; Table S7). The results showed that the expression characteristics of most genes under drought and salt stress treatments were in accordance with the gene chip data. Particularly for *MtGT20*, *MtGT22*, and *MtGT33*, they were dramatically up-regulation by drought and salt treatments at the same time. Interestingly, *MtGT-33* was remarkably up-regulated in blades at 0–2 h under drought stress but down-regulated at 2–24 h; *MtGT20* and *MtGT22* were continuously up-regulated under drought stress within 96 h. However, in salt stress treatment, *MtGT-20* and *MtGT-22* were remarkably up-regulated in blades at 2–24 h but down-regulated at 24–48 h; *MtGT33* was continuously up-regulated within 48 h. They may be involved in the response regulation of abiotic stress.

### 3.8. Expression Profiling of MtGT Genes in Response to ABA Treatments

**Figure 7:**
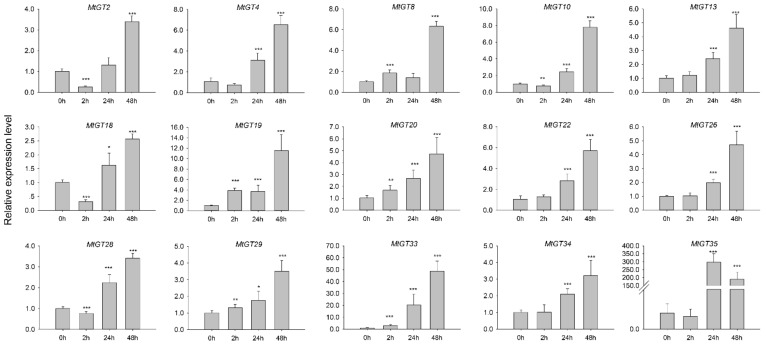
Expression profiles of 15 selected *MtGT* genes in response to ABA treatment. Expression data were normalized using *MtActin* as the internal control and error bars indicate standard deviation among three biological replicates. Asterisks indicate the corresponding genes were significantly upregulated or downregulated compared with the control (* *p* < 0.05, ** *p* < 0.01, *** *p* < 0.001; Student’s *t*-test).

Aforementioned results in this study demonstrated that some *MtGT* genes could be dramatically induced by drought and salt treatments. The phytohormone abscisic acid (ABA), which is considered as the core adversity signal in plants, plays a critical role in response to abiotic stresses such as drought, salinity, and chilling [47,48,49]. Therefore, we further verified the expression profiles of 15 differentially expressed *MtGT* genes under exogenous ABA treatment by qRT-PCR (Figure 7; Table S8). Most of the genes, particularly *MtGT19*, *MtGT33*, and *MtGT35*, had a strong response to ABA hormones. Their expression levels increased more than ten or even hundreds of times for 48 h of ABA treatment, indicating that these TFs played vital roles in response to the ABA stress signal.

## 4. Discussion

Recently, a few more trihelix TFs have been characterized which played important roles in multiple processes during plant growth and development, such as trichome development [14], shattering of the mature seed during crop domestication [17], morphogenesis control of floral organs [13], and response to abiotic and biotic stresses [15,18]. In this study, we identified 38 *MtGT* genes in *M. truncatula*, which is similar to the number of trihelix genes in *Arabidopsis* (30) [12], rice (41) [23], and tomato (36) [24]. Additionally, it is close to the average number of trihelix genes on each subgenome of wheat (31) [29]. The 38 *MtGT* genes were divided into five clades (GT-1, GT-2, SH4, GTγ, and SIP1) by constructing an unrooted phylogenetic tree to analyze and compare with trihelix family members in *Arabidopsis*. At present, the functions of several trihelix family genes, such as *PETAL LOSS* (*PTL*) gene [13], *ARABIDOPSIS 6B-INTERACTING PROTEIN1-LIKE1* (*ASIL1*) and *ASIL2* gene [50], have been studied in depth in *Arabidopsis*. It is helpful to find some *MtGT* genes which have similar functions as those reported in *Arabidopsis* through phylogenetic analysis.

We further analyzed the gene structure and conserved motifs of the trihelix family members in *M. truncatula*, and the result was consistent with the family classification. We found that most *MtGT* genes in GT-1, GT-2, and SH4 clades contained the trihelix domain (GT domain), whereas all the members of SIP1 and GTγ subfamilies contained MYB DNA-binding domain. It is consistent with the hypothesis that the trihelix domain originated from a *MYB*-like gene carrying only one repeat [5]. The most members classifying into the same clade shared similar motif compositions and exon/intron, which indicated that the specific conserved motif may play an important role in the function of a particular cluster. Among five clades, most members of GT-1 and GT-2 clades shared motifs 1, 4, 5, and 7, which had higher homology between them than that in other subfamilies of *M. truncatula*. This is similar to the results in *Arabidospsis*, and several *AtGT* genes in the GT1 and GT2 clusters have similar functions. *GT-1* and *GT-3a* of GT1 clade, *GT-2* and *DF1-like* of GT2 clade are involved in light-induced response [5,9,10,11,51]; *EMB2746* (GT1) and *EDA31* (GT2) were identified as essential for *Arabidopsis* embryo development [52,53]. As the largest subfamily, the composition of motifs of the SIP1 (most of the members of this clade shared motifs 1, 2, 3, and 4) was quite different from that of other subfamily members, whose composition of motifs was similar to that in cabbage [25], chrysanthemum [28], and wheat [29]. The functions of SIP1 members may be more complex and diverse in the trihelix family of *M. truncatula*.

Gene duplication including tandem, segmental, and genomic duplication have significant impacts on the generation of novel genes and functional diversity, facilitating the evolution and expansion of gene families in plant genomes [25,45]. There were three groups of *MtGT* genes with tandem duplication events and two pairs of *MtGT* genes with segmental duplication in trihelix genes family of *M. truncatula*. Compared with soybean (67) [27], *P. trichocarpa* (56) [30], and *B. Rapa* (52) [25], the number of *MtGT* genes with duplication events were fewer in *M. truncatula*. We speculated that most trihelix family genes originate from the different ancestors and are less conservative in *M. truncatula*. Additionally, these results indicated that gene functions of the *M. truncatula* trihelix family may have a high degree of divergence and diversity.

The tissue-expression pattern is an important factor in the study of gene functional characteristics. Based on the RNA-seq data combined with qRT-PCR verification, we can hypothesize that MtGT TFs play specific and significant roles in the growth and development of *M. truncatula*. Most of the *MtGT* genes presented a tissue-specific expression pattern in *M. truncatula*. Particularly, *MtGT12* was specifically expressed in flowers and seedpods, and *MtGT6* from GT-2 clade exhibited relatively high expression in flowers, which may affect the development of *M. truncatula* floral organs and embryoid. The *PETAL LOSS* (*PTL*) gene encoding a GT-2 TF repressed growth in the sepal whorl in *Arabidopsis*. The *ptl* mutants exhibited missing petals and partial fusion of sepal whorl [13]. The *EDA31*, a close member of *PTL*, was found to be involved in embryo sac development due to defective polar fusion in its mutant [53]. *MtGT4* and *MtGT35* were expressed at high levels in roots and nodules, and *MtGT31* and *MtGT32* were only expressed highly in nodules. This is consistent with the nature of *M. truncatula* as a typical legume with developed root system and strong nitrogen fixation ability. Tissue-specific genes may play vital roles in the growth and differentiation of the corresponding organs or tissues, but further experiments are needed to verify the biological function of these *MtGT* genes.

Recent studies have reported that trihelix genes are involved in the ABA signalling pathway in response to plant abiotic stresses. The accumulation of ABA causes stomatal closure in guard cells to prevent water loss and regulates the expression of numerous genes to induce various cellular and molecular events, such as second messenger Ca^2+^ signal system and antioxidant enzyme system, to improve stress tolerance [54,55,56]. Yu et al., reported that *ShCIGT* belonging to the GT-1 subfamily was involved in the regulation of abiotic stresses resistance in tomato by interacting with a mediator of ABA signal: SNF1-related protein kinase 1 (SnRK1) [57]. Additionally, previous studies have shown that trihelix TFs were involved in Ca^2+^ signal regulation in response to abiotic stress. *AtGT-3b* was dramatically induced by salt stress and could interact with the GT-1 cis-element of the SCaM-4 (CaM isoform) promoter in soybean responding to various environmental stresses [18]. Overexpression of *AtGT2L* which act as a Ca^2+^-dependent CaM-binding protein involved in plant stress response enhanced the tolerance to cold and salt stress in *Arabidospsis* [58]. Furthermore, overexpression of *Arabidopsis* SIP1 clade trihelix 1 (*AST1*) could regulate the expression of multiple physiological response genes, including proline biosynthesis genes, LEA family genes, *POD* and *SOD* genes, improved drought and salt stress tolerance [59]. Based on the aforementioned results, among MtGT family genes, *MtGT10*, *MtGT19*, *MtGT20*, *MtGT22*, and *MtGT33* were significantly induced by drought, salt, and ABA treatments, demonstrating their important roles in abiotic stresses response and resistance in *M. truncatula*. This study provides a valuable reference gene resource for the molecular genetic improvement of stress resistance in legumes, particularly in alfalfa. The way they participate in the ABA signalling response or interact with the Ca^2+^ signal pathway, regulatory pathways they are involved in and their role (positive or negative), and functional genes they interact with need to be further investigated.

## Figures and Tables

**Table 1:** The related information of trihelix genes in *M. truncatula*.

Name	Gene ID	Length (aa)	Signature Domain	MW (KDa)	Subcellular Localization	pI	GRAVY
*MtGT-1*	Medtr1g009220.1	319	19–104	37,270.73	Nucleus	5.36	−1.039
*MtGT-2*	Medtr1g037650.1	189	35–122	21,313.82	Nucleus	9.14	−0.917
*MtGT-3*	Medtr1g060990.1	511	129–217	58,779.54	Nucleus	6.57	−1.173
*MtGT-4*	Medtr1g061640.1	496	121–247	56,941.93	Nucleus	6.46	−1.242
*MtGT-5*	Medtr1g081180.1	557	101–181	64,373.39	Nucleus	5.71	−1.104
*MtGT-6*	Medtr1g492790.1	501	112–200	57,848.24	Nucleus	5.99	−0.921
*MtGT-7*	Medtr1g094045.1	381	53–183	42,978.72	Extracell/Nucleus	9.09	−1.072
*MtGT-8*	Medtr1g098870.1	565	53–140	64,378.61	Nucleus	5.91	−1.088
*MtGT-9*	Medtr1g098900.1	637	55–142	70,838.09	Nucleus	5.77	−0.900
*MtGT-10*	Medtr1g098920.1	450	123–250	51,386.17	Nucleus	6.16	−1.049
*MtGT-11*	Medtr1g112650.1	316	40–131	35,249.58	Chloroplast/Nucleus	4.97	−0.653
*MtGT-12*	Medtr2g016140.1	332	20–114	37,437.82	Chloroplast	5.37	−0.703
*MtGT-13*	Medtr2g016140.2	317	20–114	35,816.88	Chloroplast/Nucleus	5.29	−0.805
*MtGT-14*	Medtr2g026725.1	379	260–348	42,411.51	Nucleus	7.74	−0.924
*MtGT-15*	Medtr2g092960.1	1223	43–136	140,855.87	Nucleus	8.51	−0.845
*MtGT-16*	Medtr2g098080.1	445	112–238	50,604.51	Nucleus	6.21	−0.991
*MtGT-17*	Medtr2g102227.1	496	272–360	57,303.69	Nucleus	4.48	−1.395
*MtGT-18*	Medtr3g014280.1	325	35–122	37,084.54	Nucleus	9.03	−1.057
*MtGT-19*	Medtr3g089020.1	867	773–857	96,355.08	Chloroplast	7.97	−0.400
*MtGT-20*	Medtr3g085960.1	390	74–163	44,613.12	Chloroplast/Nucleus/Peroxisome	5.90	−0.834
*MtGT-21*	Medtr4g015680.1	279	40–125	33,950.09	Nucleus	6.55	−1.283
*MtGT-22*	Medtr4g079960.1	313	28–123	35,566.23	Nucleus	9.53	−0.778
*MtGT-23*	Medtr4g079960.2	304	28–123	34,456.87	Nucleus	9.60	−0.818
*MtGT-24*	Medtr4g079960.3	315	28–123	35,784.64	Nucleus	9.73	−0.774
*MtGT-25*	Medtr4g117990.1	340	41–135	37,889.76	Nucleus	9.07	−0.915
*MtGT-26*	Medtr5g017500.1	229	20–115	26,637.29	Nucleus	9.22	−0.834
*MtGT-27*	Medtr5g026540.1	302	43–128	35,678.99	Nucleus	8.34	−1.208
*MtGT-28*	Medtr6g035315.1	526	117–243	59,762.71	Nucleus	6.05	−0.988
*MtGT-29*	Medtr6g035370.1	646	53–140	72,115.32	Nucleus	5.74	−1.018
*MtGT-30*	Medtr6g486270.1	370	127–218	42,750.57	Nucleus	4.59	−1.147
*MtGT-31*	Medtr7g020870.1	341	38–130	37,377.48	Nucleus	5.32	−0.740
*MtGT-32*	Medtr7g020870.2	245	38–131	26,785.93	Nucleus	5.77	−0.636
*MtGT-33*	Medtr7g068770.1	293	51–136	35,338.67	Nucleus	6.62	−1.346
*MtGT-34*	Medtr7g081190.1	388	43–140	43,393.75	Nucleus	9.64	−0.969
*MtGT-35*	Medtr7g103390.1	455	101–227	52,293.03	Nucleus	6.02	−1.204
*MtGT-36*	Medtr7g114860.1	483	379–476	55,781.34	Nucleus	6.34	−0.832
*MtGT-37*	Medtr8g022290.1	371	65–161	40,319.8	Nucleus	9.53	−0.803
*MtGT-38*	Medtr8g100130.1	361	45–139	40,525.42	Nucleus	5.36	−0.803

Length: Protein length (aa); MW: Protein molecular weight (kDa); pI: isoelectric point; GRAVY: Grand average of hydropathicity.

## Supplementary Materials

The following are available online at https://www.mdpi.com/2073-4425/11/11/1389/s1, Table S1: Nucleotide and amino acid sequences of 38 MtGTs. Table S2: Conserved amino acid sequences of motifs in MtGTs. Table S3: The collinearity relationships of the *MtGT* genes with five species. Table S4: The relative FPKM value of 36 *MtGT* genes in different tissues. Table S5: Expression levels of nine *MtGT* genes in different tissues by qRT-PCR. Table S6: Expression data of 33 *MtGT* gene chips in the blades under drought and salt treatments. Table S7: Expression levels of nine *MtGT* genes under drought and salt treatments by qRT-PCR. Table S8: Expression data of fifteen *MtGT* genes under exogenous ABA treatment. Table S9: Sequences of 21 gene-specific primer pairs used for qRT-PCR validation. Figure S1: The multiple sequence alignment of the MtGT and AtGT proteins.

## References

[1] Wray G.A., Hahn M.W., Abouheif E., Balhoff J.P., Pizer M., Rockman M.V., Romano L.A. (2003). The evolution of transcriptional regulation in eukaryotes. Mol. Biol. Evol..

[2] Riechmann J.L., Heard J., Martin G., Reuber L., Jiang C., Keddie J., Adam L., Pineda O., Ratcliffe O.J., Samaha R.R. (2000). *Arabidopsis* transcription factors: Genome-wide comparative analysis among eukaryotes. Science.

[3] Spitz F., Furlong E.E.M. (2012). Transcription factors: From enhancer binding to developmental control. Nat. Rev. Genet..

[4] Zhou D.X. (1999). Regulatory mechanism of plant gene transcription by GT-elements and GT-factors. Trends Plant Sci..

[5] Nagano Y. (2000). Several features of the GT-factor trihelix domain resemble those of the Myb DNA-binding domain. Plant Physiol..

[6] Gilmartin P.M., Memelink J., Hiratsuka K., Kay S.A., Chua N.H. (1992). Characterization of a gene encoding a DNA binding protein with specificity for a light-responsive element. Plant Cell.

[7] Green P.J., Kay S.A., Chua N.H. (1987). Sequence-specifc interactions of a pea nuclear factor with light-responsive elements upstream of the *rbcS-3A* gene. EMBO J..

[8] Perisic O., Lam E. (1992). A tobacco DNA binding protein that interacts with a light-responsive box II element. Plant Cell.

[9] Hiratsuka K., Wu X., Fukuzawa H., Chua N.H. (1994). Molecular dissection of GT-1 from *Arabidopsis*. Plant Cell.

[10] Maréchal E., Hiratsuka K., Delgado J., Nairn A., Qin J., Chait B.T., Chua N.H. (1999). Modulation of GT-1 DNA-binding activity by calcium-dependent phosphorylation. Plant Mol. Biol..

[11] Dehesh K., Smith L.G., Tepperman J.M., Quail P.H. (1995). Twin autonomous bipartite nuclear localization signals direct nuclear import of GT-2. Plant J..

[12] Kaplan-Levy R.N., Brewer P.B., Quon T., Smyth D.R. (2012). The trihelix family of transcription factors - light, stress and development. Trends Plant Sci..

[13] Brewer P.B., Howles P.A., Dorian K., Griffith M.E., Ishida T., Kaplan-Levy R.N., Kilinc A., Smyth D.R. (2004). *PETAL LOSS*, a trihelix transcription factor gene, regulates perianth architecture in the *Arabidopsis* flower. Development.

[14] Breuer C., Kawamura A., Ichikawa T., Tominaga-Wada R., Wada T., Kondou Y., Muto S., Matsui M., Sugimoto K. (2009). The trihelix transcription factor GTL1 regulates ploidy-dependent cell growth in the *Arabidopsis* trichome. Plant Cell.

[15] Yoo C.Y., Pence H.E., Jin J.B., Miura K., Gosney M.J., Hasegawa P.M., Mickelbart M.V. (2010). The *Arabidopsis* GTL1 transcription factor regulates water use efficiency and drought tolerance by modulating stomatal density via transrepression of *SDD1*. Plant Cell.

[16] Gao M.J., Lydiate D.J., Li X., Lui H., Gjetvaj B., Hegedus D.D., Rozwadowski K. (2009). Repression of seed maturation genes by a trihelix transcriptional repressor in *Arabidopsis* seedlings. Plant Cell.

[17] Lin Z., Griffith M.E., Li X., Zhu Z., Tan L., Fu Y., Zhang W., Wang X., Xie D., Sun C. (2007). Origin of seed shattering in rice (*Oryza sativa* L.). Planta.

[18] Park H.C., Kim M.L., Kang Y.H., Jeon J.M., Yoo J.H., Kim M.C., Park C.Y., Jeong J.C., Moon B.C., Lee J.H. (2004). Pathogen-and NaCl-induced expression of the SCaM-4 promoter is mediated in part by a GT-1 box that interacts with a GT-1-like transcription factor. Plant Physiol..

[19] Zheng X., Liu H., Ji H., Wang Y., Dong B., Qiao Y., Liu M., Li X. (2016). The wheat GT factor *TaGT2L1D* negatively regulates drought tolerance and plant development. Sci. Rep..

[20] Fang Y., Xie K., Hou X., Hu H., Xiong L. (2010). Systematic analysis of GT factor family of rice reveals a novel subfamily involved in stress responses. Mol. Genet. Genomics.

[21] Young N.D., Debellé F., Oldroyd G.E.D., Geurts R., Cannon S.B., Udvardi M.K., Benedito V.A., Mayer K.F.X., Gouzy J., Schoof H. (2011). The *Medicago* genome provides insight into the evolution of rhizobial symbioses. Nature.

[22] Cañas L.A., Beltrán J.P. (2018). Model legumes: Functional genomics tools in *Medicago truncatula*. Methods Mol. Biol..

[23] Li J., Zhang M., Sun J., Mao X., Wang J., Wang J., Liu H., Zheng H., Zhen Z., Zhao H. (2019). Genome-wide characterization and identification of trihelix transcription factor and expression profiling in response to abiotic stresses in rice (*Oryza sativa* L.). Int. J. Mol. Sci..

[24] Yu C., Cai X., Ye Z., Li H. (2015). Genome-wide identification and expression profiling analysis of trihelix gene family in tomato. Biochem. Biophys. Res. Commun..

[25] Wang W., Wu P., Liu T.K., Ren H., Li Y., Hou X. (2017). Genome-wide analysis and expression divergence of the trihelix family in *Brassica Rapa*: Insight into the evolutionary patterns in plants. Sci. Rep..

[26] Mo H., Wang L., Ma S., Yu D., Lu L., Yang Z., Yang Z., Li F. (2019). Transcriptome profiling of *Gossypium arboreum* during fiber initiation and the genome-wide identification of trihelix transcription factors. Gene.

[27] Osorio M.B., Bücker-Neto L., Castilhos G., Turchetto-Zolet A.C., Wiebke-Strohm B., Bodanese-Zanettini M.H., Margis-Pinheiro M. (2012). Identification and in silico characterization of soybean trihelix-GT and bHLH transcription factors involved in stress responses. Genet. Mol. Biol..

[28] Song A., Wu D., Fan Q., Tian C., Chen S., Guan Z., Xin J., Zhao K., Chen F. (2016). Transcriptome-wide identification and expression profiling analysis of chrysanthemum trihelix transcription factors. Int. J. Mol. Sci..

[29] Xiao J., Hu R., Gu T., Han J., Qiu D., Su P., Feng J., Chang J., Yang G., He G. (2019). Genome-wide identification and expression profiling of trihelix gene family under abiotic stresses in wheat. BMC Genomics.

[30] Wang Z., Liu Q., Wang H., Zhang H., Xu X., Li C., Yang C. (2016). Comprehensive analysis of trihelix genes and their expression under biotic and abiotic stresses in *Populus trichocarpa*. Sci. Rep..

[31] Zhang H., Jin J., Tang L., Zhao Y., Gu X., Gao G., Luo J. (2011). PlantTFDB 2.0: Update and improvement of the comprehensive plant transcription factor database. Nucleic Acids Res..

[32] El-Gebali S., Mistry J., Bateman A., Eddy S.R., Luciani A., Potter S.C., Qureshi M., Richardson L.J., Salazar G.A., Smart A. (2019). The Pfam protein families database in 2019. Nucleic Acids Res..

[33] Lu S., Wang J., Chitsaz F., Derbyshire M.K., Geer R.C., Gonzales N.R., Gwadz M., Hurwitz D.I., Marchler G.H., Song J.S. (2020). CDD/SPARCLE: The conserved domain database in 2020. Nucleic Acids Res..

[34] Letunic I., Bork P. (2018). 20 years of the SMART protein domain annotation resource. Nucleic Acids Res..

[35] Wilkins M.R., Gasteiger E., Bairoch A., Sanchez J.C., Williams K.L., Appel R.D., Hochstrasser D.F. (1999). Protein identification and analysis tools in the ExPASy server. Methods Mol. Biol..

[36] Chou K.C., Shen H.B. (2010). Plant-mPLoc: A top-down strategy to augment the power for predicting plant protein subcellular localization. PLoS ONE.

[37] Chen C., Chen H., Zhang Y., Thomas H.R., Frank M.H., He Y., Xia R. (2020). TBtools: An integrative toolkit developed for interactive analyses of big biological data. Mol. Plant.

[38] Wang Y., Tang H., Debarry J.D., Tan X., Li J., Wang X., Lee T., Jin H., Marler B., Guo H. (2012). MCScanX: A toolkit for detection and evolutionary analysis of gene synteny and collinearity. Nucleic Acids Res..

[39] Bailey T.L., Elkan C. (1994). Fitting a mixture model by expectation maximization to discover motifs in biopolymers. Proc. Int. Conf. Intell. Syst. Mol. Biol..

[40] Waterhouse A.M., Procter J.B., Martin D.M.A., Clamp M., Barton G.J. (2009). Jalview Version 2—A multiple sequence alignment editor and analysis workbench. Bioinformatics.

[41] Kumar S., Stecher G., Li M., Knyaz C., Tamura K. (2018). MEGA X: Molecular evolutionary genetics analysis across computing platforms. Mol. Biol. Evol..

[42] Subramanian B., Gao S., Lercher M.J., Hu S., Chen W.H. (2019). Evolview v3: A webserver for visualization, annotation, and management of phylogenetic trees. Nucleic Acids Res..

[43] He J., Benedito V.A., Wang M., Murray J.D., Zhao P.X., Tang Y., Udvardi M.K. (2009). The *Medicago truncatula* gene expression atlas web server. BMC Bioinform..

[44] Livak K.J., Schmittgen T.D. (2001). Analysis of relative gene expression data using real time quantitative PCR and the 2^−ΔΔCT^ method. Methods.

[45] Innan H., Kondrashov F. (2010). The evolution of gene duplications: Classifying and distinguishing between models. Nat. Rev. Genet..

[46] Ma Z., Liu M., Sun W., Huang L., Wu Q., Bu T., Li C., Chen H. (2019). Genome-wide identification and expression analysis of the trihelix transcription factor family in tartary buckwheat (*Fagopyrum tataricum*). BMC Plant Biol..

[47] Kim T.H., Böhmer M., Hu H., Nishimura N., Schroeder J.I. (2010). Guard cell signal transduction network: Advances in understanding abscisic acid, CO_2_, and Ca^2+^ signaling. Annu. Rev. Plant Biol..

[48] Cutler S.R., Rodriguez P.L., Finkelstein R.R., Abrams S.R. (2010). Abscisic acid: Emergence of a core signaling network. Annu. Rev. Plant Biol..

[49] Umezawa T., Nakashima K., Miyakawa T., Kuromori T., Tanokura M., Shinozaki K., Yamaguchi-Shinozaki K. (2010). Molecular basis of the core regulatory network in ABA responses: Sensing, signaling and transport. Plant Cell Physiol..

[50] Willmann M.R., Mehalick A.J., Packer R.L., Jenik P.D. (2011). MicroRNAs regulate the timing of embryo maturation in *Arabidopsis*. Plant Physiol..

[51] Ayadi M., Delaporte V., Li Y.F., Zhou D.X. (2004). Analysis of GT-3a identifies a distinct subgroup of trihelix DNA-binding transcription factors in *Arabidopsis*. FEBS Lett..

[52] Tzafrir I., Pena-Muralla R., Dickerman A., Berg M., Rogers R., Hutchens S., Sweeney T.C., McElver J., Aux G., Patton D. (2004). Identification of genes required for embryo development in *Arabidopsis*. Plant Physiol..

[53] Pagnussat G.C., Yu H.J., Ngo Q.A., Rajani S., Mayalagu S., Johnson C.S., Capron A., Xie L.F., Ye D., Sundaresan V. (2005). Genetic and molecular identification of genes required for female gametophyte development and function in *Arabidopsis*. Development.

[54] Fujii H., Chinnusamy V., Rodrigues A., Rubio S., Antoni R., Park S.Y., Cutler S.R., Sheen J., Rodriguez P.L., Zhu J.K. (2009). In vitro reconstitution of an abscisic acid signalling pathway. Nature.

[55] Raghavendra A.S., Gonugunta V.K., Christmann A., Grill E. (2010). ABA perception and signalling. Trends Plant Sci..

[56] Munemasa S., Hauser F., Park J., Waadt R., Brandt B., Schroeder J.I. (2015). Mechanisms of abscisic acid-mediated control of stomatal aperture. Curr. Opin. Plant Biol..

[57] Yu C., Song L., Song J., Ouyang B., Guo L., Shang L., Wang T., Li H., Zhang J., Ye Z. (2018). *ShCIGT*, a trihelix family gene, mediates cold and drought tolerance by interacting with SnRK1 in tomato. Plant Sci..

[58] Xi J., Qiu Y., Du L., Poovaiah B.W. (2012). Plant-specific trihelix transcription factor *AtGT2L* interacts with calcium/calmodulin and responds to cold and salt stresses. Plant Sci..

[59] Xu H., Shi X., He L., Guo Y., Zang D., Li H., Zhang W., Wang Y. (2018). *Arabidopsis thaliana* trihelix transcription factor AST1 mediates abiotic stress tolerance by binding to a novel AGAG-box and some GT motifs. Plant Cell Physiol..

