# Supplementary figures and images for: Genome-Wide Identification and Expression Profiling Analysis of the Trihelix Gene Family Under Abiotic Stresses in Medicago truncatula
Source: Genes (Basel). 2020 Nov 23;11(11):1389. doi: 10.3390/genes11111389 (PMC7709032; doi:10.3390/genes11111389)

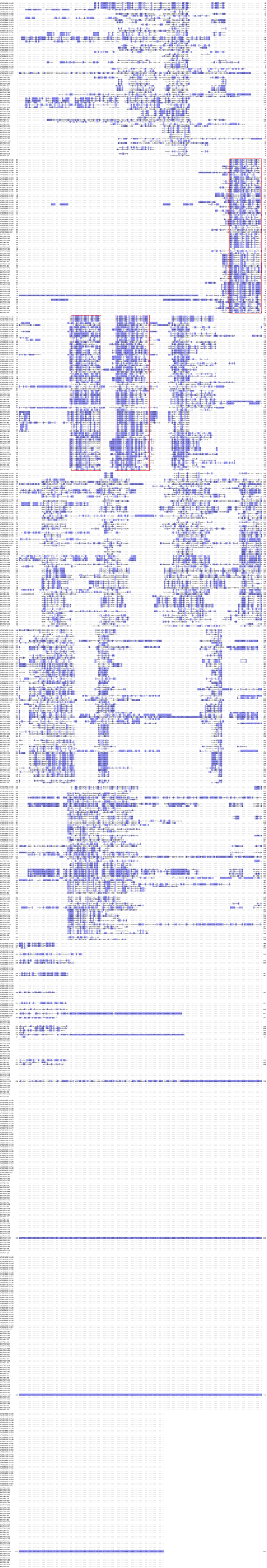

Supplement: Supplementary file 1 [file genes-11-01389-s001.zip › genes-992818-supplementary/supplementary files/Figure S1.png]
